# Secondary Hyperparathyroidism in Children with Mucolipidosis Type II (I-Cell Disease): Irish Experience

**DOI:** 10.3390/jcm11051366

**Published:** 2022-03-02

**Authors:** Ritma Boruah, Ahmad Ardeshir Monavari, Tracey Conlon, Nuala Murphy, Andreea Stroiescu, Stephanie Ryan, Joanne Hughes, Ina Knerr, Ciara McDonnell, Ellen Crushell

**Affiliations:** 1 National Centre for Inherited Metabolic Diseases (NCIMD), Children’s Health Ireland at Temple Street, D01 XD99 Dublin, Ireland; ahmad.monavari@cuh.ie (A.A.M.); joanne.hughes@cuh.ie (J.H.); ina.knerr@cuh.ie (I.K.); ellen.crushell@cuh.ie (E.C.); 2 School of Medicine, University College Dublin, D04 V1W8 Dublin, Ireland; tracey.conlon@cuh.ie (T.C.); nuala.murphy@cuh.ie (N.M.); 3 Department of Endocrinology, Children’s Health Ireland at Temple Street, D01 XD99 Dublin, Ireland; ciara.mcdonnell@cuh.ie; 4 Department of Radiology, Children’s Health Ireland at Temple Street, D01 XD99 Dublin, Ireland; andreeastroiescu@gmail.com (A.S.); stephanie.ryan99@gmail.com (S.R.)

**Keywords:** mucolipidosis type II, ML II, I-cell disease, hyperparathyroidism

## Abstract

Mucolipidosis type II (ML II) is an autosomal recessive lysosomal targeting disorder that may present with features of hyperparathyroidism. The aim of this study was to describe in detail the clinical cases of ML II presenting to a tertiary referral centre with biochemical and/or radiological features of hyperparathyroidism. There were twenty-three children diagnosed with ML II in the Republic of Ireland from July 1998 to July 2021 inclusive (a 23-year period). The approximate incidence of ML II in the Republic of Ireland is, therefore, 1 per 64,000 live births. Medical records were available and were reviewed for 21 of the 23 children. Five of these had been identified as having biochemical and/or radiological features of hyperparathyroidism. Of these five, three children were born to Irish Traveller parents and two to non-Traveller Irish parents. All five children had radiological features of hyperparathyroidism (on skeletal survey), with evidence of antenatal fractures in three cases and an acute fracture in one. Four children had biochemical features of secondary hyperparathyroidism. Three children received treatment with high dose Vitamin D supplements and two who had antenatal/acute fractures were managed with minimal handling. We observed resolution of secondary hyperparathyroidism in all cases irrespective of treatment. Four of five children with ML II and hyperparathyroidism died as a result of cardiorespiratory failure at ages ranging from 10 months to 7 years. Biochemical and/or radiological evidence of hyperparathyroidism is commonly identified at presentation of ML II. Further studies are needed to establish the pathophysiology and optimal management of hyperparathyroidism in this cohort. Recognition of this association may improve diagnostic accuracy and management, facilitate family counseling and is also important for natural history data.

## 1. Introduction

Mucolipidosis type II (ML II) (OMIM #252500) or inclusion cell disease (I-cell disease) is a rare autosomal recessive lysosomal enzymetargeting disease due to deficiency of uridine diphosphate-N-acetylglucosamine: lysosomal enzyme N-acetylglucosamine-1-phosphotransferase (GlcNac-1-phosphotransferase). This enzyme is involved in the first step of the mannose 6-phosphate signal, which allows specific targeting of lysosomal acid hydrolase from the trans-Golgi network to lysosomes. The enzyme deficiency precludes the generation of the common phosphomannosyl recognition marker of lysosomal enzymes [1]. Subsequently, newly synthesized lysosomal enzymes are secreted into the extracellular space rather than targeted to the lysosomes. Thus, affected lysosomes are secondarily deficient in most acid hydrolases; undigested junk materials accumulate within the lysosomes [1,2]. ML II was first described as inclusion-cell (I-cell) disease by Leroy and Demars in 1967 [3], because the fibroblasts derived from patients contain abundant ‘inclusions’ (now recognized as swollen lysosomes) within the cytoplasm. These inclusions are observed not only in cultured skin fibroblasts, but also in a variety of other cell types in vivo, including peripheral blood lymphocytes [2].

The term “mucolipidosis” was introduced in 1970 by Spranger and Wiedemann to describe several conditions with features both of mucopolysaccharidoses (MPS) and sphingolipidoses [4]. ML II is a progressive multi-organ disease, usually with prenatal clinical onset and fatal outcome within the first decade of life due to cardiopulmonary complications [5]. It is characterized by coarse facial features, short stature, hyperplastic gums, organomegaly, retarded psychomotor development and skeletal deformities, which may include shortened limbs, flexion contractures and talipes [6,7]. Secondary hyperparathyroidism is a recognized feature of ML II [1,7,8,9,10,11]. Reported biochemical features of secondary hyperparathyroidism include elevated parathyroid hormone (PTH), serum calcium (Ca), alkaline phosphatase (ALP) levels and low levels of phosphate (P) [8,11,12,13,14], Radiographic findings in neonates resemble changes of rickets and/or hyperparathyroidism. These changes include osteopenia, subperiosteal resorption, poor cortical delineation, periosteal new bone formation with ‘cloaking’ (linear periosteal new bone parallel to the shaft of the bone but widely separated from the bone), metaphyseal irregularity and submetaphyseal lucent bands and later develop into Hurler-type dysostosis multiplex. Congenital long bone and rib fractures are rare and likely the result of severe osteopenia and disorganized bone formation [9,12,15,16,17]. Bone changes can precede elevations in biochemical markers [12]; therefore, regular monitoring in infancy is required and skeletal radiographs should be performed regardless of initial biochemical findings.

Clinical suspicion is the first step in establishing a diagnosis of ML II, with typical clinical features often apparent at birth or otherwise manifesting in the first year of life [12]. An indirect diagnosis is usually established by measurement of lysosomal hydrolases, both in white blood cells, where their levels should be low, and their surrounding extracellular environment (e.g., plasma), where their levels should be high [18]. The diagnosis is confirmed by *GNPTAB* gene molecular analysis; this is particularly important in cases when biochemical testing is inconclusive or carrier detection is required [11,19]. There are at least 258 mutations reported in the *GNPTAB* gene, the most prevalent being c. 3503_3504del. Despite increased prevalence of homozygous mutations, particularly in highly consanguineous populations, the autosomal recessive inheritance of MLII means a high number of compound heterozygous *GNPTAB* sequence alterations [5]. ML II is a multi-ethnic disease. It has been identified in many different ethnic groups [9,11,19,20,21,22,23,24,25], with reported prevalence as follows: Portugal—approximately 1:123, 500 live births [22], Japan—1:252, 500 live births [23] and 1:625, 500 live births in Netherlands [24].

ML II has a very high incidence of 1 per 909 live births in the Irish Traveller community [26]. Irish Travellers are an endogamous grouup who have cultural values and customs quite distinct from that of the “settled community”, i.e., the non-Traveller Irish population. Cultural traditions within the community include a preference to marry within their own community (often resulting in consanguineous unions), young age at marriage and large families [26].

Hyperparathyroidism is not universal but has been observed in patients with MLII [1,7,8,9,10,11,12,14,27]. The biochemical and radiological features of hyperparathyroidism in infants with ML II vary in the literature [9,10,12,15,16,27,28], with resolution of these findings observed in many cases, even in the absence of active management [14,17,27]. It is known that, following this initial early period where features of hyperparathyroidism or rickets may be observed, children with ML II experience a progressive osteodystrophy [9]. Recognition and active management of hyperparathyroidism may prevent complications such as bone fractures and, thus, improve quality of life for the affected children. It is also important to recognize hyperparathyroidism in this cohort for natural history data. Here, we describe clinical, biochemical, radiological and molecular findings in five children with ML II and hyperparathyroidism from 5 unrelated families.

## 2. Materials and Methods

This study was performed in The National Centre for Inherited Metabolic Diseases, Children’s Health Ireland (CHI) at Temple Street in the Republic of Ireland. A retrospective chart review of ML II patients born between July 1998 and July 2021 inclusive was performed, providing a twenty-three-year cohort. A database was compiled documenting clinical features, focusing on those consistent with hyperparathyroidism. For those diagnosed with hyperparathyroidism, biochemical, radiological and molecular data were recorded. This study was approved by the Research and Ethics Committee of CHI at Temple Street (protocol code 21. 013).

## 3. Results

We identified 23 patients with ML II from 14 families, born between 1 July 1998 and 1 July 2021. This gives an approximate national incidence of 1 per 64,000 live births in the Republic of Ireland. Medical records were available for 21 out of 23 children. Of the 23 identified, 19 were from the Irish Traveller community, confirming the very high incidence within the Traveller community, in line with previously reported figure of 1 in 909 [26]. In this cohort, five patients from five families had biochemical and/or radiological evidence of hyperparathyroidism, three of these children were born to Irish Traveller parents.

### 3.1. Diagnosis of ML II—Biochemical and Molecular Genetic Features

The diagnosis of ML II was based on clinical features and biochemical testing by detecting increased activity of alpha mannosidase and beta hexosaminidase in plasma. In all cases, the diagnosis was confirmed by molecular genetic testing; these patients were found to be homozygous for a common mutation c.3503_3504delTC (p.L1168Qfs*5) in *GNPTAB* gene.

### 3.2. Biochemical Features of Hyperparathyroidism

All five patients had been tested for hyperparathyroidism within the first few weeks of life and four had increased levels of parathyroid hormone (PTH). Calcium (Ca) and phosphate (P) levels were normal; however, alkaline phosphatase (ALP) levels were markedly raised in four of five cases (Patients 2, 3, 4, 5). Vitamin D levels were checked in four cases (Patients 2, 3, 4, 5) and were normal in three, with one (Patient 5) having a suboptimal level of 30 nmol/L (normal range is >50 nmol/L).

### 3.3. Radiological Abnormalities Identified

**Figure 1:**
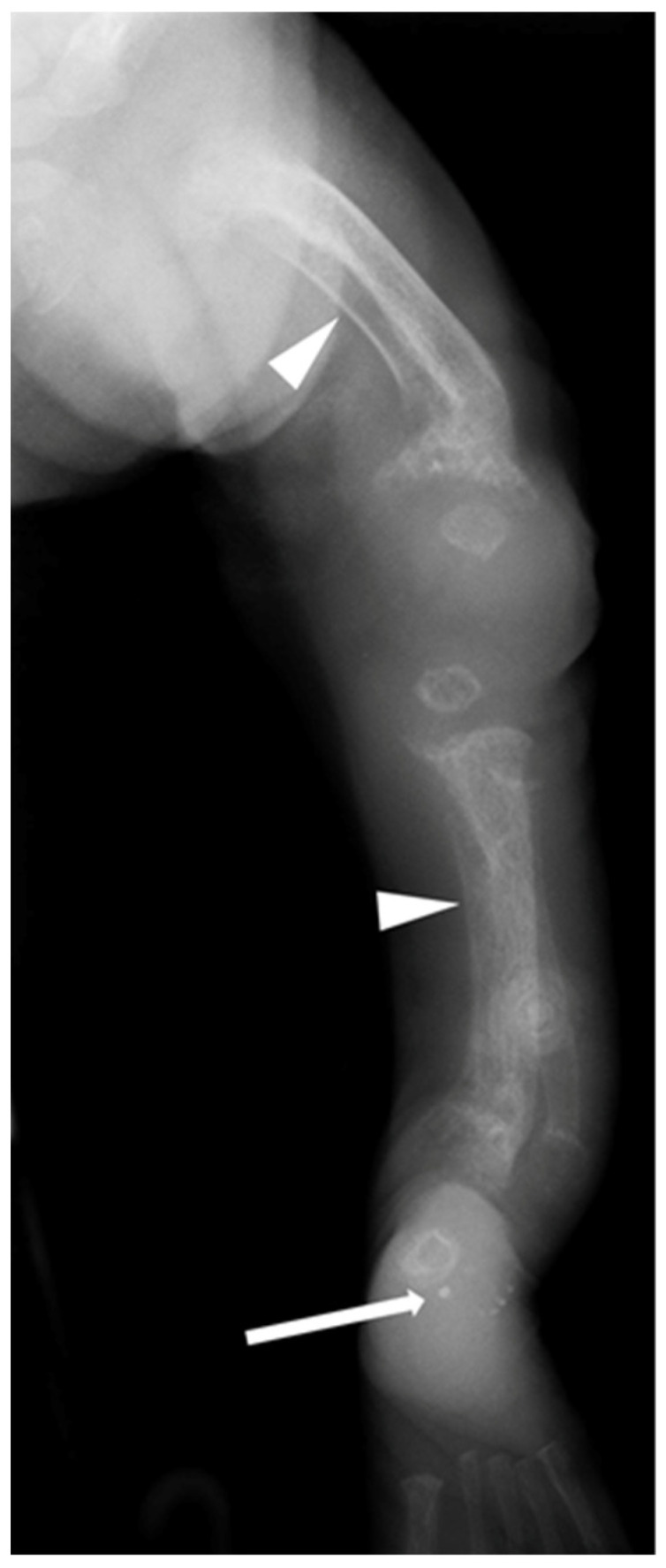
Acute findings of hyperparathyroidism and rickets. Patient 1, left leg radiograph day 1 demonstrates features of hyperparathyroidism including reduced bone density, subperiosteal resorption and poor cortical delineation (see medial tibia) as well as diaphyseal cloaking of the femur and tibia (arrowheads). Features of rickets are seen with cupped, splayed and frayed metaphyses especially in the distal femur. Diaphyseal angulation consistent with antenatal fractures is seen in the distal femur and tibia. Additionally, talocalcaneal stippling, a feature of I-cell disease is also present (white arrow).

**Figure 2:**
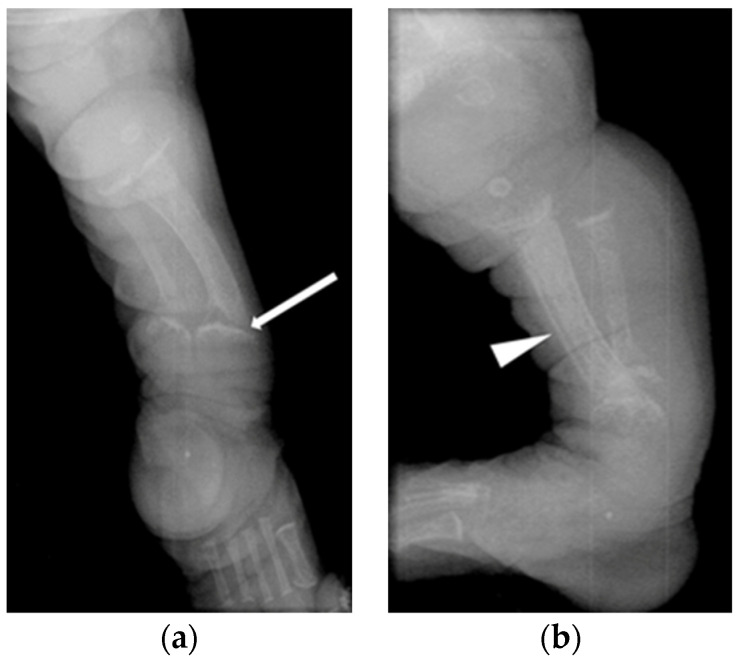
Acute findings of hyperparathyroidism and of rickets. Patient 2, right leg radiograph day 4 (**a**) frontal and (**b**) lateral views show features of hyperparathyroidism (best seen on the lateral view) including reduced bone density, subperiosteal resorption and poor cortical delineation as well as diaphyseal cloaking of the tibia (arrowheads). Features of rickets are seen with cupped, splayed and frayed metaphyses in all the bones. A submetaphyseal lucent band is seen in the tibia (white arrow). Talocalcaneal stippling is also present.

Skeletal radiographs in the first week of life were available for four patients and at 2 months of age for one patient (Patient 3). Follow up radiographs were available for four out five patients. Early radiographs already showed marked changes of hyperparathyroidism in all five infants including osteopenia, subperiosteal resorption, poor cortical delineation, periosteal new bone formation with ‘cloaking’ (linear periosteal new bone parallel to the shaft of the bone but widely separated from the bone), metaphyseal irregularity and submetaphyseal lucent bands (Figure 1 and Figure 2).

**Figure 3:**
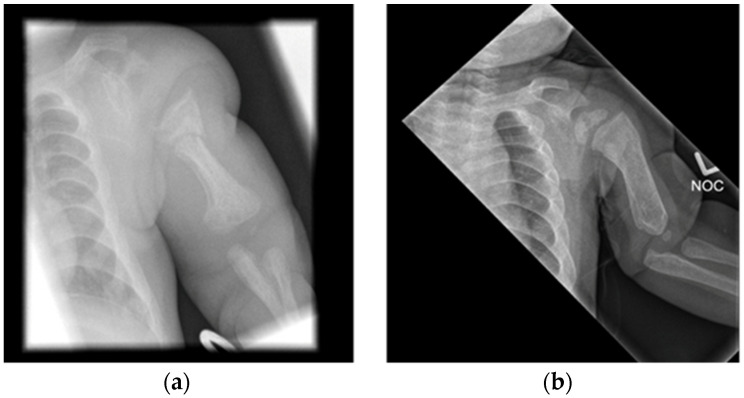
Acute fracture with healing at 4 months. Patient 4 (**a**) radiograph of left humerus on day 1 shows a transverse fracture of the left humerus. (**b**) Follow-up radiograph 4 months later showing interval healing of the fracture and resolution of the periosteal cloaking. There are already emerging features of dysostosis multi-plex with widening of the shaft and short length of the humerus and coarse trabecular markings as well as thickening of the ribs.

All early radiographs also showed features of rickets, with metaphyseal cupping, fraying and splaying (Figure 1 and Figure 2). There was evidence of antenatal long bone fractures in Patients 1, 2 and 4. Patient 4 also had an acute fracture of the proximal left humeral neck (Figure 3). Further radiographic findings of ML II including talocalcaneal stippling were identified in Patients 1, 2 and 5, and an abnormal appearance of the vertebral bodies with increased height; rounding and sclerosis were seen in Patients 1, 2 and 4.

**Figure 4:**
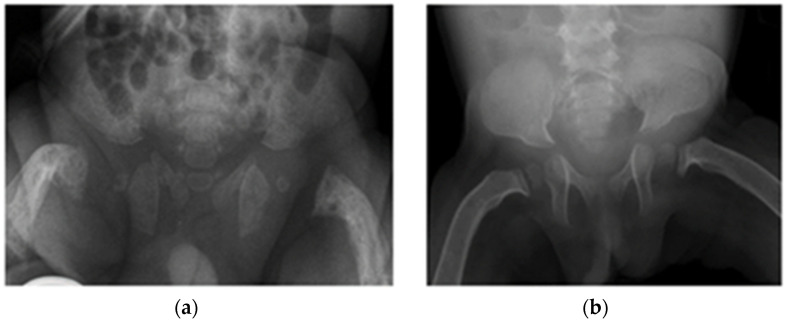
Progression to dysostosis multiplex. Patient 4 (**a**) Radiograph of pelvis on day 1 shows a reduced bone density and irregularity of the proximal femora with periosteal cloaking. (**b**) Follow-up radiograph at 2 years old shows normal bone density. The pelvis now has a typical shape of dysostosis multiplex with constriction of the lower part of the iliac bones. There has been interval healing of the rickets of the proximal femora and resolution of the periosteal cloaking.

Follow-up radiographs in all but the most recent patient showed resolution of the features of hyperparathyroidism and rickets, with interval healing of the fractures. There was progression of skeletal features to those of dysostosis multiplex, the constellation of radiographic abnormalities classically seen in mucopolysaccharidoses (MPS), including coarse trabecular markings, broadening of the ribs (oar/paddle shaped ribs), flared iliac wings, constricted inferior iliac bodies and dysplastic femoral heads (Figure 3 and Figure 4).

**Figure 5:**
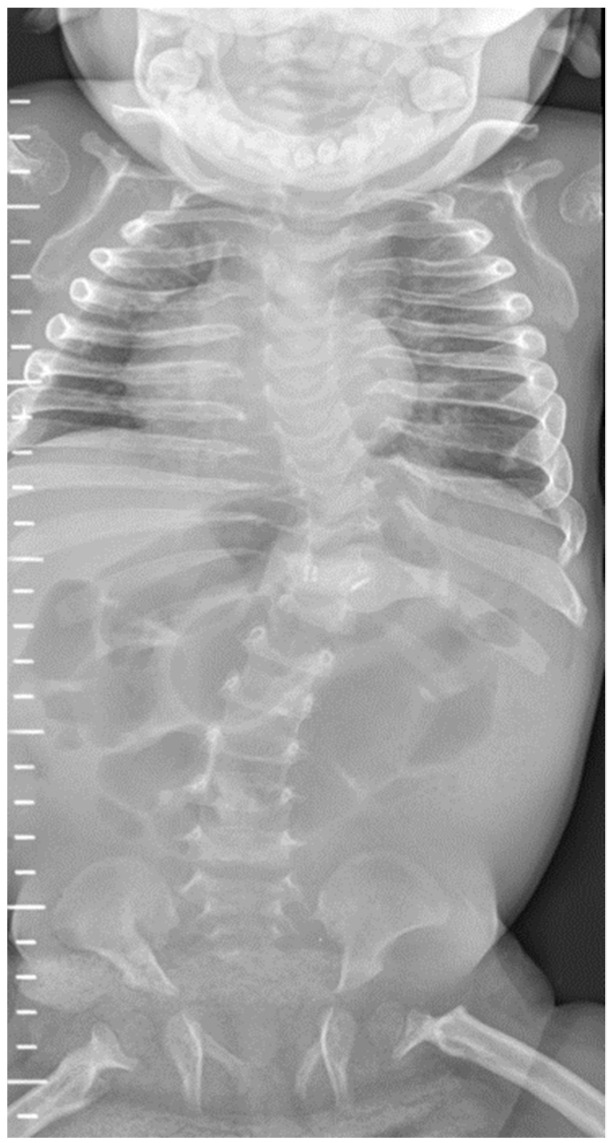
Progressive erosive osteodystrophy in Patient 1 at 6 years. Resolution of the acute changes seen in Figure 1. Development of a progressive erosive osteodystrophy with erosion of the heads and necks of the ribs, erosion of the lower part of the iliac bones, erosion of the ischial and pubic bones and of the femoral necks.

Follow up radiographs in Patient 1 showed resolution of the acute changes, including the periosteal reaction and subperiosteal erosion, but development of a progressive erosive osteodystrophy with erosion of the humeral and femoral necks and also erosion of the necks of the ribs was observed (Figure 5).

### 3.4. Management and Clinical Course

Two of the infants who had evidence of antenatal fractures (Patients 1, 4), including the one with an acute fracture (Patient 1), received treatment with increased Vitamin D supplementation of 600 IU per day and guidance around minimal handling, including a lie flat car seat, lying supported on the side and avoidance of walkers and bouncers. In the case of Patient 4, the previously abnormal biochemical parameters normalized within five months of treatment with vitamin D; the dose was then reduced to the standard supplementation dose of 200 IU/day, with handling liberalized successfully. A follow-up left humeral radiograph 4 months later showed interval healing of the humeral neck fracture (Figure 3). Patient 1 was followed up at a local hospital and vitamin D dose was reduced to 200 IU/day and handling liberalized at 1 year.

Patients 2 and 3 were monitored without specific treatment. In Patient 2, PTH level had spontaneously returned to normal at 10 months. In the case of Patient 3, the previously abnormal biochemical marker (ALP) had normalized at 10 months of age (during an admission to Pediatric Intensive Care unit with respiratory failure). No further fractures were observed. Patient 5 was started on high dose Vitamin D supplementation of 1000 IU/day with interval re-evaluation planned.

Four of the five affected infants died from progressive cardiopulmonary decline at ages ranging from 10 months to 7 years. Summary of clinical, biochemical, radiological features of secondary hyperparathyroidism, Vitamin D levels at diagnosis and treatment can be found in Table 1.

## 4. Discussion

Patients with ML II have been reported to present in the neonatal period with features of ‘’metabolic’’ bone disease [9,27]. While features of dysostosis multiplex (the constellation of radiographic abnormalities classically seen in mucopolysaccharidoses) are seen in older children with ML II, the skeleton in ML II in the young infant is characterized by an osteodystrophy which has clinical and radiographic features of hyperparathyroidism and rickets and these changes have been reported in ML II as early as 19 weeks of gestation [28].

Osteoporosis, fractures, periosteal new bone formation and cupped epiphyses have been described in neonates and infants [9]. Radiological and histological features of both rickets and hyperparathyroidism (subperiosteal bone resorption and loss of bone mass) have been documented in babies with I-cell disease [15,16]. Biochemical evidence of hyperparathyroidism is more variable but had been reported in some affected neonates [9].

In our study, none of the patients had abnormal serum calcium or phosphate levels. Similar findings were reported by David-Vizcarra et al. [9]. Four out of five of our patients had increased levels of ALP and PTH. All children had early skeletal x-rays with radiographic evidence of hyperparathyroidism, including osteopenia, subperiosteal bone resorption, poor cortical delineation, periosteal new bone formation, metaphyseal irregularity and submetaphyseal lucent bands. Three had evidence of antenatal fractures. All early radiographs also had features of rickets, with metaphyseal cupping, fraying and splaying.

Sathasivam et al. [10] previously described similar findings in a female neonate with neonatal hyperparathyroidism and rickets-like radiographic changes. Alfadhel et al. [11] reported two children with raised PTH and ALP, normal calcium levels who also had radiographic changes consistent with rickets/hyperparathyroidism. Some authors speculate that the presence of severe skeletal changes related to secondary hyperparathyroidism indicate that the abnormal elevation of PTH starts in utero [8].

The exact etiology of secondary hyperparathyroidism in ML II remains unclear. It has been suggested that the active transplacental transport of calcium is interrupted by ML II. The syncytiotrophoblastic layer where active transplacental calcium transport is regulated demonstrates generalized cytoplasmic vacuolization in patients with ML II. This suggests that the enzymatic abnormalities related to ML II interfere in some way with transplacental calcium transport. PTH secretion is thought to increase to maintain extracellular calcium at the expense of the skeleton [27]. This, however, does not explain skeletal changes in those with normal PTH levels.

David-Vizcarra et al. [9] observed that, following birth, biochemical hyperparathyroidism in ML II resolves, but a progressive erosive osteodystrophy develops after 4 months of age. We saw this progressive erosive osteodystrophy in one patient whose early PTH levels were not increased. They proposed that tissue hypersensitivity to circulating PTH (“pseudohyperparathyroidism”) may be a factor. They confirmed that circulating levels of parathyroid related protein (PTHrP) were normal and postulated that, postnatally, the radiographic features could be consistent with an increased sensitivity of skeletal tissue to normal circulating levels of PTH. A more recent study by Kollmann et al. [29], however, refuted tissue hypersensitivity to PTH as a pathogenetic mechanism for the osteopenia, since the secretion of Rankl (pro-osteoclastogenic cytokine) in osteoblasts from ML II mice was not affected in response to PTH stimulation.

The above demonstrates the need for further studies regarding the pathophysiology of bone disease in patients with ML II.

Optimal management of the secondary hyperparathyroidism in this patient cohort is also controversial, as changes may be self-limiting and might represent the natural history of ML II [14,17,27].

Unger et al. [27] described three patients with bone disease, increased serum PTH and ALP, but normal calcium levels. Two were treated with Vitamin D and calcium supplements, while one received no treatment and secondary hyperparathyroidism resolved in all cases. Another report by Leyva et al. [8] described a patient with secondary hyperparathyroidism (with biochemical and radiographic changes), in whom a spontaneous normalization of previously elevated PTH was observed in the absence of treatment, however the ALP level remained high and radiographic follow up was not reported.

In contrast, a patient reported by Khan et al. [12] had low serum phosphate, normal vitamin D and PTH levels at birth and radiographic findings of rickets/hyperparathyroidism. Initially, after parents declined vitamin D supplementation, ALP and PTH levels rose significantly. At 4 months of age, parents agreed to vitamin D supplementation and, within a month, serum phosphate, PTH and ALP levels normalized. This indicates that bone disease can precede the elevations in biochemical markers and highlights the importance of regular biochemical monitoring, even if initial PTH levels are normal. We recommend checking for biochemical and radiographic features of secondary hyperparathyroidism at diagnosis of ML II by checking levels of PTH, Ca, P, ALP, Vitamin D and by performing skeletal survey. We suggest monitoring of biochemical markers at least 6–12 monthly during infancy; however, such monitoring intervals are arbitrary and would depend on the initial levels. Similarly, the frequency of radiological monitoring would depend on initial clinical and radiographic findings, e.g., sooner re-imaging for those with bone fractures. Rapid normalization of biochemical markers post commencement of vitamin D supplementation may indicate that this supplement has a role in the treatment of secondary hyperparathyroidism in some patients with ML II. Currently, in our centre, we recommend high dose Vitamin D supplements in those with biochemical features of secondary hyperparathyroidism and/or bone fractures and low/suboptimal Vitamin D levels. Minimal handling, including a lie flat car seat, lying supported on the side and avoidance of walkers and bouncers would be recommended for children with bone fractures.

Antiresorptive therapy has also been suggested as a therapeutic option, but this is usually reserved for individuals with a high fracture risk and this treatment is controversial in ML II, given the multisystem involvement and overall poor prognosis [29].

Unger et al. [27] summarized that most children with a neonatal presentation of ML II and perinatal bone disease have a shortened life expectancy, i.e., most die before the age of 2 years. In our cohort with secondary hyperparathyroidism, all patients had perinatal bone disease confirmed by skeletal survey and two died before the age of 2 years.

Given the rarity of ML II, extensive sequential assessment of five patients with secondary hyperparathyroidism from a clinical, radiological and biochemical perspective provides important information regarding natural history of the condition. Our paper also provides recommendations on diagnostics and management of secondary hyperparathyroidism in this cohort; we believe that these would help to improve diagnostic accuracy and to optimize management of these patients. In addition, our paper contains interesting figures depicting clinical and radiological course of the disease and provides an up-to-date incidence of ML II in Republic of Ireland.

While we identified five members of the cohort as having secondary HPT, not all infants were systematically investigated for the same; therefore, it is likely that there is under-ascertainment.

## 5. Conclusions

Children with ML II may have varying degrees of radiological and biochemical features of hyperparathyroidism at presentation. It is important to recognize this association, as this may improve diagnostic accuracy and management. It is also important for appropriate family counseling and natural history data. In this cohort, resolution of abnormal biochemical findings was observed in all cases, irrespective of management. Further studies are needed to establish the etiology and pathophysiology of the bony changes observed in ML II and the potential benefit of Vitamin D supplementation in this cohort.

## Figures and Tables

**Table 1:** Summary Summary of clinical, biochemical, radiological features of secondary hyperparathyroidism and Vitamin D levels at diagnosis and treatment.

	Clinical Features	PTH Reference Range: 11–25 ng/L	Ca Reference Range: 2.15–2.65 mmol/L	P Reference Range: 1.2–2.0 mmol/L	ALP Reference Range: 60–550 IU/L	Vitamin D Reference Range: >50 nmol/L	Radiological Features	Treatment
Patient 1	Antenatal fractures of long bones	25	2.46	1.65	434	Not available in medical notes	All patients had features of HPT (Figure 1 and Figure 2), including: Osteopenia Subperiosteal resorption Poor cortical delineation Periosteal new bone formation with ‘cloaking’ (linear periosteal new bone parallel to the shaft of the bone but widely separated from the bone) Metaphyseal irregularity Submetaphyseal lucent bands	Vitamin D 600 IU, minimal handling until 1 year of age
Patient 2	Antenatal fractures of long bones	119 ↑	2.36	1.17	1540 ↑	66.7		None
Patient 3	No fractures	72 ↑	2.45	1.96	1063 ↑	48 (reference range > 15 mmol/L)		None
Patient 4	Acute fracture of the proximal left humeral neck (Figure 3a), antenatal fractures	252 ↑	2.32	1.62	1007 ↑	121		Vitamin D 600 IU, minimal handling for 5 month
Patient 5	No fractures	110 ↑	2.31	1.3	1256 ↑	30 ↓		Vitamin D 1000 IU, re-evaluation planned in due course

↑ raised level; ↓ reduced level.

## Data Availability

The data presented in this study are available on request from the corresponding author. The data are not publicly available due to privacy restrictions.
